# Coxa vara with proximal femoral growth arrest as a possible consequence of extracorporeal membrane oxygenation: a case report

**DOI:** 10.4076/1757-1626-2-8130

**Published:** 2009-08-11

**Authors:** Juan Pretell Mazzini, Juan Rodríguez Martín, Rafael Marti Ciruelos

**Affiliations:** Department of Orthopaedic Surgery, 12 Octubre HospitalAv. Córdoba, s/n Madrid28041 Spain

## Abstract

Coxa vara is an abnormality of the proximal femur with a decreased neck-shaft angle and resulting leg-length discrepancy, has been associated with conditions such as congenital coxa vara, traumatic injury, sepsis, rickets, vascular damage, or metabolic disorders; however its possible relationship with extracorporeal membrane oxygenation has been recently reported.

A full term girl was born with a total infradiaphragmatic anomalous pulmonary venous drainage, at the age of 12 days, an anastomosis of the pulmonary venous trunk with the left auricle and closure of the auricular septal defect was performed; during this procedure extracorporeal membrane oxygenation was used during 104 minutes, no neonatal sepsis was developed. She had no orthopedic issues until she was 3 years and 10 months old, when she presented with limp related to the right lower limb, with no pain. She had a leg-length discrepancy of 2 cm (right - left), limited right hip abduction to 25°, and internal rotation to 5°, also had a positive Trendelenburg test. No flexion/extension abnormalities. Anteroposterior radiographs and magnetic resonance revealed coxa vara with proximal femoral growth arrest. A valgus osteotomy with greater trochanteric epiphysiodesis was performed. At the eight month follow-up, she had no hip pain, better hip range of motion, no difficulties with recreational activities and the osteotomy healed.

Another four similar cases had been reported with similar outcome, we think that it will be recommendable to take images in patients with this background and limb leg-length discrepancy or abnormal range of motion.

## Introduction

Coxa vara may refer descriptively to any varus deformity of the femoral neck or it may refer to more specific entities which have been commonly designed as congenital coxa vara, developmental coxa vara [1,2] and acquired coxa vara [3].

The roentgenographic appearance of coxa vara is distinctive in the AP view of the pelvis. The neck-shaft angle is less than 110-120° [1,4], the growth plate is tilted to near vertical orientation, and the greater trochanter may be elevated above the femoral head.

Clinically the patient presents with an abnormal gait and a leg length discrepancy. The limb is generally painless and a waddling gait is seen when the deformity is bilateral. The involved hip will often display limitation of abduction and internal rotation [1].

Extracorporeal membrane oxygenation (ECMO) is a form of prolonged extracorporeal cardiorespiratory bypass achieved by extrathoracic cannulation. Complications include intracranial hemorrhage, bleeding, thrombosis and neurodevelopmental delays [3,5-7].

To the best of our knowledge there are only other four similar cases reported at the English literature by one author [3]; this report confirm the characterization of this hip abnormality and presents a new possible cause of acquired coxa vara.

## Case presentation

A full term Caucasian Spanish girl was born via vaginal delivery, her birth weight was 1800 g, she had a total infradiaphragmatic anomalous pulmonary venous drainage and for that reason she was managed at the intensive care unit until the age of 12 days when an anastomosis of the pulmonary venous trunk with the left auricle and closure of the auricular septal defect was performed; during this procedure extracorporeal membrane oxygenation was used during 104 minutes, the patient did well during ECMO, the wound was clear without effusions, the platelet count was within normal range (150-400×10^3^/ml), WBC count was normal (10-20×10^3^/ml) no fever was registered. She subsequently did well and met all of her early developmental milestones, including walking at age 12 months.

She had no orthopedic issues until she was 3 year-10 months-old, when she presented with limp related to the right lower limb, with no pain. She had a leg-length discrepancy of 2 cm (right - left), limited right hip abduction to 25°, and internal rotation to 5°, also had a positive Trendelenburg test. No flexion/extension abnormalities.

**Figure 1.**
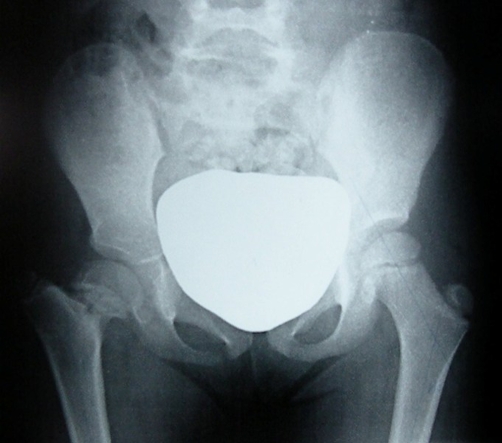
Anteroposterior radiograph of the pelvis shows coxa vara and growth arrest with the femoral neck shortening and an elevated greater trochanter.

**Figure 2.**
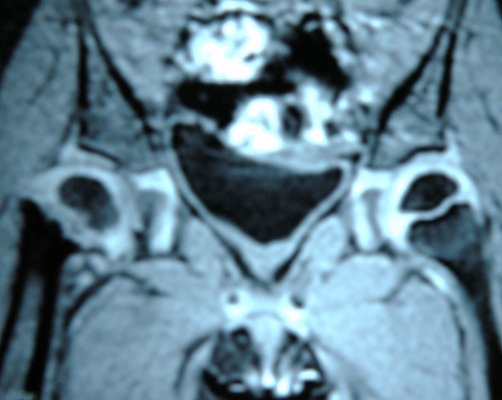
Magnetic resonance image revealed metaphyseal irregularity and physes widening.

Anteroposterior radiographs of the pelvis showed coxa vara with abnormality of the proximal femoral ossification centers and proximal femoral neck associated to an elevated greater trochanter (Figure 1), a neck-shaft angle of 100° and a HE angle of 50°. Magnetic resonance imaging revealed metaphyseal irregularities with widening of the physes (Figure 2).

Progression of the coxa vara, elevation of the greater trochanter and limitation in her recreational activities developed.

**Figure 3.**
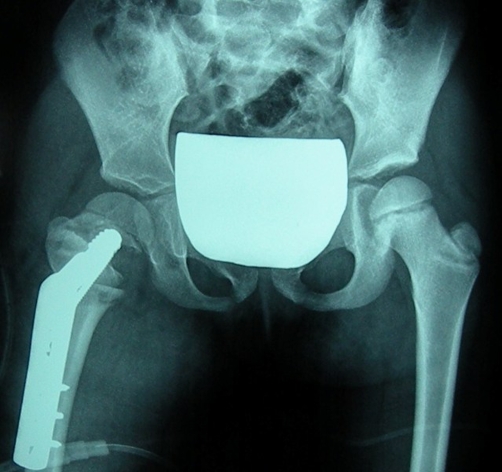
Anteroposterior radiograph of the pelvis showing the valgus osteotomy and greater trochanter epiphysiodesis.

At the age of 6 years and 5 months she underwent a right femoral valgus flexion osteotomy associated to a greater trochanteric epiphysiodesis (Figure 3). The neck-shaft angle was corrected to 140°.

**Figure 4.**
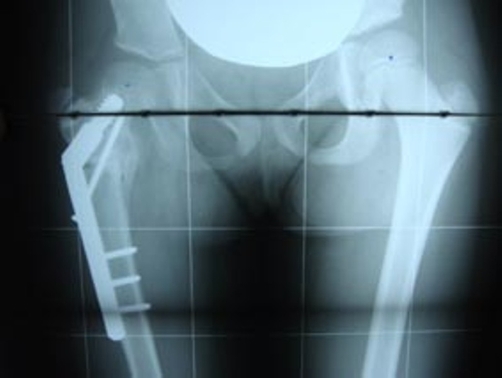
Anteroposterior pelvis radiograph demonstrates osteotomy healed.

At the eight month follow-up, she had no hip pain, near normal hip range of motion, no difficulties with recreational activities and the osteotomy healed (Figure 4).

## Discussion

We have identified another patient with a pattern of coxa vara and proximal femoral growth arrest with a history of neonatal treatment with ECMO. Our patient clinical presentation is similar to the one described by DiFazio *et al*., she had progressive gait disturbance, leg length discrepancy, limited abduction of the affected hip but differs in which no pain was present. Another difference was that ECMO was done during a shorter time through the cardiac procedure.

Coxa vara has been classified into three categories: congenital, acquired and developmental. Congenital coxa vara is caused by an embryonic limb bud abnormality and is present at birth [3], developmental coxa vara usually occurs as an isolated deformity of the proximal femur and goes unnoticed until the child reaches walking age and is brought for assessment of a leg length discrepancy or abnormal gait, and acquired coxa vara results from an underlying condition such as fibrous dysplasia, rickets, or traumatic proximal femoral epiphyseal plate closure [3], this is important because the definitive treatment depends on which type of coxa vara are we talking about.

The goal of treatment is restoration of the neck-shaft angle and reorientation of the growth plate to decrease shear forces and promote ossification of the femoral neck defect [1]. This is accomplished with a valgus osteotomy, the valgus position of the femoral neck enhances the action of the gluteal muscles, restores the angle of the neck to normal, increases the length of the limb, and may even improve the congruity of the joint [8].

Surgical indications include a neck-shaft angle less than 90°, progressive deformity, vertical physis and a significant limb [1]. Other indications are based on the HE angle, an HE angle greater than 60° is indicative of surgery, between 45-60° close follow up and less than 45° spontaneous resolution [9]. Also if the neck-shaft angle is less than 110°, the natural history is progression of the varus angulation, gait abnormalities and early degenerative changes [4].

Our patient had an HE angle of 50° however the neck-shaft angle was 100° and presented a progressive deformity and recreational activities limitations so she underwent a right valgus flexion osteotomy with epiphysiodesis of the greater trochanter. The evolution was satisfactory like the cases reported by DiFazio *et al*.

## Conclusion

These findings show that there could be a different spectrum of clinical presentation with a shorter time of ECMO.

This case report reinforces the possible association between ECMO and this pattern of coxa vara with proximal femoral growth arrest.

## Abbreviation

ECMO: extracorporeal membrane oxygenation
